# Sequence and phylogenetic analysis of the mitochondrial genome for the Wolf-eel, *Anarrhichthys ocellatus* (Anarhichadidae: Perciformes)

**DOI:** 10.1080/23802359.2019.1660260

**Published:** 2019-09-03

**Authors:** Fenghui Li, Xiangtang Chen, Gang Lu, Jiangbo Qu, Li Bian, Qing Chang, Jianlong Ge, Changlin Liu, Shengnong Zhang, Siqing Chen

**Affiliations:** a Laboratory for Marine Fisheries Science and Food Production Processes, Pilot National Laboratory for Marine Science and Technology, Yellow Sea Fisheries Research Institute, Chinese Academy of Fishery Sciences, Qingdao, China;; b National Demonstration Center for Experimental Fisheries Science Education, Shanghai Collaborative Innovation for Aquatic Animal Genetics and Breeding, Shanghai Engineering Research Center of Agriculture, Shanghai Ocean University, Shanghai, China;; c Yantai Marine Economic Research Institute, Yantai, China;; d Weihai Inspection and Testing Center for Food and Drug, Weihai, China;; e Tianyuan Aquatic Products Co., Ltd, Yantai, China

**Keywords:** mtDNA, *Anarrhichthys ocellatus*, mitochondrion, phylogenetic analysis

## Abstract

The first complete mitochondrial genome of the wolf-eel (*Anarrhichthys ocellatus*) was determined and analyzed in this work. It had a circular mapping molecular with the length of 16,506 bp and contained 12S and 16S rRNAs, 22 tRNAs, 13 protein-coding genes, and a 851 bp D-loop in the typical arrangement of the vertebrate consensus. Phylogenetic analysis of the mitochondrial genome sequences of 47 representative species within the order Perciformes suggests that *A. ocellatus* is closely related to the species in the family Anarhichadidae. *Anarrhichthys ocellatus* mitogenome can contribute to our understanding of the phylogeny and evolution of this species.

The wolffish family Anarhichadidae, a small family of perch-like marine fish, contains two genera: the *Anarrhichthys* and *Anarhichas* (Johnstone et al. 2007). The wolf-eel (*Anarrhichthys ocellatus* Ayres, 1855) is monotypic within the *Anarrhichthys*, mainly found in the north pacific distributing from the Aleutian island to southern California, the depth of which is ranged from 1 to 226 meters (Hubbs and Barnhart 1944; Willimovsky 1964), frequently deserved on the rocky and hard bottom of the marine. Due to the use of the bottom-trawling in the marine fisheries, the populations and habitats of the wolffish have been seriously impacted and the abundance declined significantly (Johnstone et al. 2007). Recently, the *A. ocellatus* is not the target of the directed fisheries and mainly used for exhibition, and the information on it is still lacked. To make a better understanding of the phylogeny and evolution of *A. ocellatus*, the complete mitochondrial DNA genome sequence was needed.

In this study, we report the first complete mitochondrial DNA genome sequence for *A. ocellatus*. A wild female *A. ocellatus* (123.8 cm standard length, 5225.3 g mass) was collected from an aquafarm in Yantai, Shandong province, China (37°42′36″N, 121°04′59″E). Tissue from this specimen was archived in the Yellow Sea Fisheries Research Institute, Chinese Academy of Fishery Sciences (Tissue #AO-201506). The genomic DNA was extracted from the muscle tissue by the standard phenol–chloroform extraction procedure (Sambrook and Russell 2001). Six primer sets were designed to amplify the segments. The Resulting PCR products were Sanger sequenced, assembled and then annotated using MitoFish (Iwasaki et al. 2013). The complete mitochondrial genome sequence of *A. ocellatus* is 16,506 bp in length with GC content of 46.18% (GenBank accession no. MG551528), containing 13 protein-coding genes (PCGs), 22 transfer RNA (tRNA) genes, two ribosomal RNA (rRNA) genes, one replication origin (OL), and one control region (D-loop). All genes show the typical gene arrangement conforming to the vertebrate consensus (Noack et al. 1996). Among the 13 protein-coding genes, except ND2, COX2, ATP6, ND3, ND4, and CYTB use an incomplete stop codon ‘T––’ or ‘TA–’, the rest are encoded by the typical TAA or TAG stop codons. The length of the control region (D-Loop) is 851 bp.

To confirm the phylogenetic position of *A. ocellatus*, the whole mitochondrial genomes of 47 species from 13 families of Order Perciformes were aligned using Clustal X software (Larkin et al. 2007), and maximum-likelihood (ML) analysis was conducted using MEGA 7.0 with 1000 bootstrap replicates (Kumar et al. 2016). The ML tree showed that *A. ocellatus* (*Anarrhichthys*) is sister to genus *Anarhichas,* which belong to the family Anarhichadidae (Figure 1).

**Figure 1.**
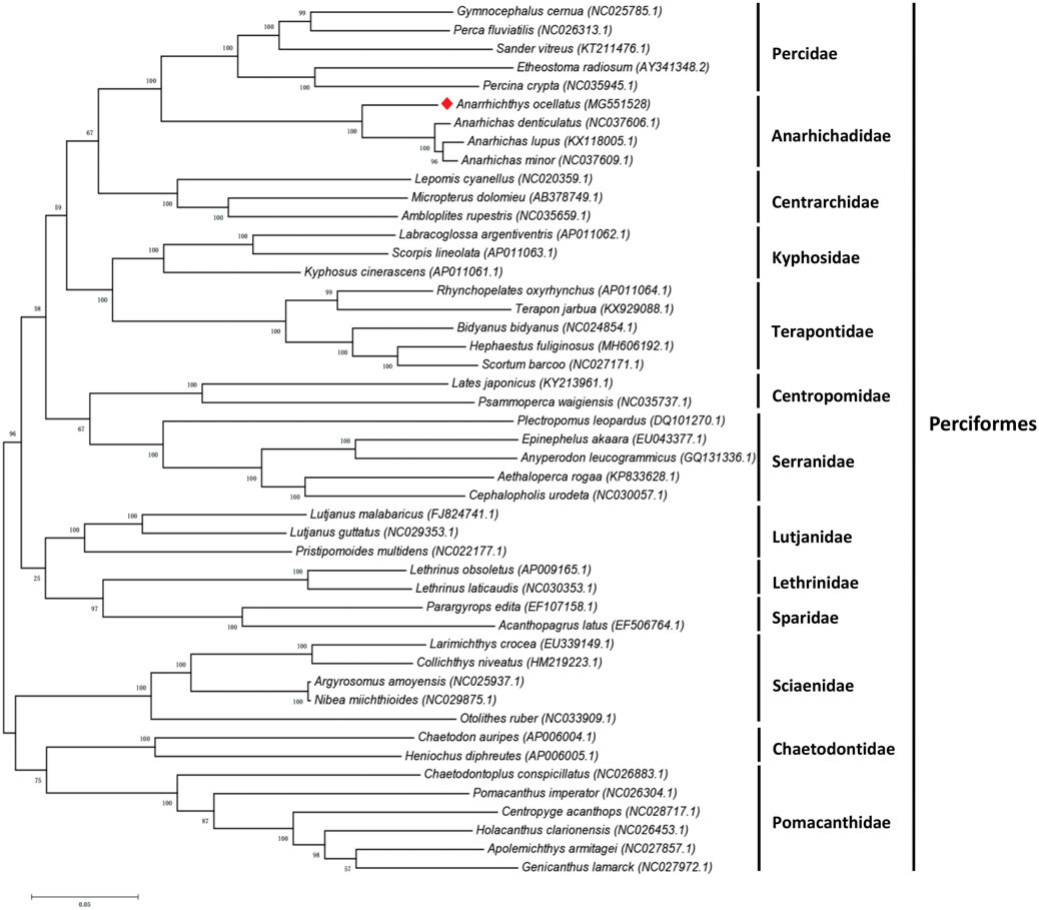
The maximum-likelihood phylogenetic tree of the complete mitogenome of *A. ocellatus* (marked with red rhombus, GeneBank accession no. MG551528) and other 46 fishes of Order Perciformes. Bootstrap support is indicated for each branch.
